# Identification of a newly described OsHV-1 µvar from the North Adriatic Sea (Italy)

**DOI:** 10.1099/jgv.0.001042

**Published:** 2018-03-26

**Authors:** Miriam Abbadi, Gianpiero Zamperin, Michele Gastaldelli, Francesco Pascoli, Umberto Rosani, Adelaide Milani, Alessia Schivo, Emanuele Rossetti, Edoardo Turolla, Lorenzo Gennari, Anna Toffan, Giuseppe Arcangeli, Paola Venier

**Affiliations:** ^1^​ Istituto Zooprofilattico Sperimentale Delle Venezie, Legnaro (PD), Italy; ^2^​ Department of Biology, University of Padova, Padova (PD), Italy; ^3^​ Consorzio Cooperative Pescatori del Polesine, Scardovari (RO), Italy; ^4^​ Istituto Delta Laboratorio CRIM, Goro (FE), Italy; ^5^​ Bivi s.r.l., Civitanova Marche (MC), Italy

**Keywords:** *Crassostrea gigas*, *Ostreid herpesvirus* type 1 μVar, OsHV-1-PT, next generation sequencing, *de novo* assembly, complete genome

## Abstract

The surveillance activities for abnormal bivalve mortality events in Italy include the diagnosis of *ostreid herpesvirus* type 1 (OsHV-1) in symptomatic oysters. OsHV-1-positive oysters (*Crassostrea gigas*) were used as a source for *in vivo* virus propagation and a virus-rich sample was selected to perform shotgun sequencing based on Illumina technology. Starting from this unpurified supernatant sample from gills and mantle, we generated 3.5 million reads (2×300 bp) and *de novo* assembled the whole genome of an Italian OsHV-1 microvariant (OsHV-1-PT). The OsHV-1-PT genome encodes 125 putative ORFs, 7 of which had not previously been predicted in other sequenced *Malacoherpesviridae*. Overall, OsHV-1-PT displays typical microvariant OsHV-1 genome features, while few polymorphisms (0.08 %) determine its uniqueness. As little is known about the genetic determinants of OsHV-1 virulence, comparing complete OsHV-1 genomes supports a better understanding of the virus pathogenicity and provides new insights into virus–host interactions.

*Ostreid herpesvirus* type 1 (OsHV-1) was firstly detected from *Crassostrea gigas* larvae during mortality events in French hatcheries in 1991 [1, 2] and it was then progressively associated with the mass mortality of oyster spats and juveniles in Europe and other regions of the world [3–8].

More aggressive OsHV-1 indicated as microvariants of the reference virus [9] (GenBank ID: AY509253) have increasingly been detected since 2008 in Europe (France, Ireland, United Kingdom, Netherlands, Spain, Portugal and Italy), as well as in other parts of the world, and at present they likely represent the prevailing OsHV-1 virus type [7, 10–13]. Most of the sequence data referring to OsHV-1 refer to diagnostic genome regions (i.e. ORF4, ORF36-37-38, ORF42-43, ORF88, ORF99 and ORF100) [4, 12, 14–17]. Up to now, the genomes of seven *Malacoherpesviridae* have been sequenced and comparatively reported, namely the reference OsHV-1 [9], two OsHV-1 µVars [13] and four OsHV-1 variants detected in other bivalve species [18–20] (KU096999, NCBI April 2016).

Italy is the third European producer of marine bivalves, with more than 100 000 tons estimated in 2015 [21]. The bivalve farming industry is economically relevant for the regions bordering the Adriatic Sea and, even if the production of the Pacific oyster is at its onset, the global diffusion of infectious OsHV-1 microvariants raises significant concern in the national authorities and farmers.

In this study, we investigated the identity and infectivity of an OsHV-1 virus detected in oysters, diploid *C. gigas*, produced and farmed in the North Adriatic Sea. Basically, a first supernatant from pooled virus-positive oysters allowed us to propagate the virus in nine subsequent *in vivo* infection trials, from which we selected a homogenate of pooled gills and mantle to purify total DNA and sequence the whole genome of a new OsHV-1 microvariant, applying next-generation sequencing and *de novo* assembly protocols (Fig. S1, available in the online version of this article).

In April 2016, initial signs of mortality were observed following an event of reduced water salinity (25 psu; 18–19 °C) in oysters no more than 4–5 months of age, farmed in the Porto Tolle area (Po Delta basin, North Adriatic Sea, Italy). No massive mortality appeared, but some moribund and asymptomatic individuals were found to be positive for the presence of OsHV-1 DNA and were used as a source for the subsequent inocula (Table 1). Details on the DNA extraction and quantitative real-time (qPCR) protocols are reported in the Supplementary Materials and methods. Following experimental infection models based on the intramuscular injection of OsHV-1 preparations [22–25], we set up an infection protocol that aimed to produce and maintain a suitable quantity of virus *in vivo*, in the absence of mollusk cell lines, and to characterize the Porto Tolle OsHV-1, hereafter referred to as OsHV-1-PT. A batch of about 300 native *C. gigas* of about 4 cm in shell length and 4–5 months of age, obtained from the Porto Tolle area (Consorzio Cooperative Pescatori del Polesine, Scardovari), was preliminarily demonstrated to be OsHV-1-negative through the testing of 30 individuals with the standard qPCR protocol that was then used to measure the OsHV-1 DNA in the injected oysters. As detailed in the Methods section (see also the Supplementary Materials and methods), up to 13 oysters per trial were tentatively infected (145 in total), while the negative controls (10 per trial) were injected with the same volume of sterile seawater. At the end of each trial, the gills and mantle [25 mg wet weight (w.w.) tissue] were sampled from individual oysters to assess the presence of OsHV-1 DNA (ORF100 region) by qPCR. Starting from the naturally infected oysters, all inocula were freshly prepared by homogenization of the pooled gills and mantle fragments of oysters showing viral titres above 10^6^ OsHV-1 copies µl^−1^. Native OsHV-1-free oysters were experimentally injected with a minimum viral load of 10^7^ DNA copies. In the first infection trial (I), the injection of 2.5–2.8×10^8^ OsHV-1 DNA copies caused 50 % mortality. In the subsequent eight infection trials (II–IX), the injection of 1.0×10^7^–1.0×10^10^ OsHV-1 DNA copies caused lower levels of oyster mortality (0–41.4 %). Relating the copy number of OsHV-1 DNA of each inoculum to the copy number of OsHV-1 DNA detected in moribund oysters, the greatest mortality levels were found to be associated with inocula with 1×10^8^ or more OsHV-1 DNA copies. Variable viral DNA titres were detected in both dead (0–1.8×10^8^ viral copies µl^−1^) and surviving oysters (0–4.9×10^7^ viral copies µl^−1^) (Table 1).

**Table 1.** Description of the nine virus propagation trials. The oyster numbers per trial, together with the volume and viral load of the inoculum and the final viral load in the injected oysters are reported

Trial	No. of injected oysters	Inoculum vol. (µl)	Injected viral load (DNA copies)	Detected viral load (copies µl^−1^)
1	13	100	2.5×10^8^–2.8×10^8^	0–2.4×10^7^
2	12	100	2.9×10^8^–1×10^9^	0–1×10^8^
3	13	150	1×10^10^	0–8.3×10^6^
4	13	100	1.5×10^8^–3.3×10^8^	0–1.2×10^6^
5*	12	150	1.2×10^8^–3.8×10^8^	0–7.7×10^7^
5†	10	150	1.2×10^8^–3.8×10^8^	0–7.7×10^7^
6	13	100	2.2×10^7^–7.7×10^9^	5.6×10^2^–1.8×10^5^
7*	13	150	1×10^7^–3.3×10^9^	0–1.8×10^8^
7†	10	150	1×10^7^–3.3×10^9^	0–1.8×10^8^
8*	13	100	1.7×10^9^	4.1×10^2^–1.9×10^6^
8†	10	100	1.7×10^9^	4.1×10^2^–1.9×10^6^
9	13	100	1×10^7^–1×10^10^	0–4.9×10^7^

* and † indicate oysters injected and kept in separate tanks.

Using the Kaplan–Meier method (reported in detail in the Supplementary Materials and methods) we estimated the oyster survival probability over time, which was found to be 97.3 % on the first day and 75 % on the sixth day post-injection, while no mortality was observed in the control animals (Fig. 1).

**Fig. 1.**
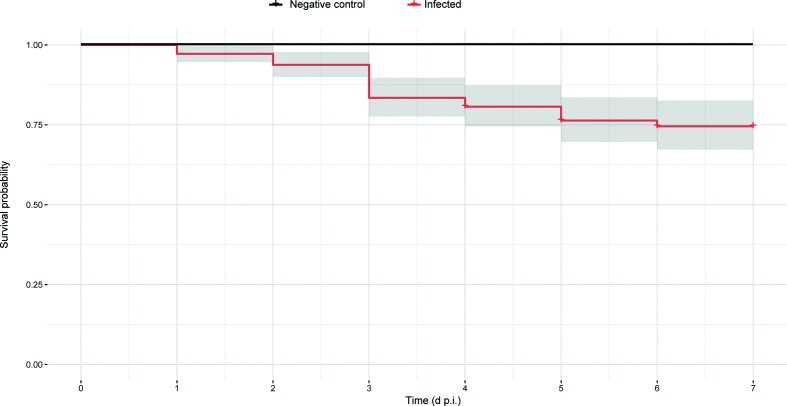
Cumulative Kaplan–Meier curves describing the survival probability of oysters infected with OsHV-1-PT.

Amongst the samples generated during the nine infection trials, we selected one sample that was rich in OsHV-1-PT (1.8×10^8^ copies µl^−1^) for confirmatory transmission electron microscopy (TEM). Tissues were prepared according to standard procedures, negatively stained with 2 % sodium phosphotungstate solution and finally observed via TEM (Philips 208S). Virions that were compatible with herpesvirus particles in terms of both size and shape were detected (Fig. S2).

The relative amounts of OsHV-1 DNA and *C. gigas* DNA in such a virus-rich sample were assessed by qPCR with the same set of primers used for the OsHV-1 DNA quantification and with a primer set designed for elongation factor (EF1α), a single-copy oyster gene (see details in Supplementary Materials and methods). The resulting ratio of 25 : 1 copy number between OsHV-1 and *C. gigas* made us confident in applying a direct next-generation sequencing (NGS) approach to the total DNA, purified from the above-mentioned virus-positive supernatant, without a virus purification step.

Following library preparation and Illumina sequencing, we generated 3 436 820 paired-end reads (2×300 bp), which allowed the recovery of 87 582 high-quality reads truly belonging to the order *Herpesvirales* (2.6 % OsHV-1 DNA to exogenous DNA ratio). The OsHV-1 reads represented a 200-fold base pair sequence coverage and a 279-fold physical coverage of the OsHV-1 genome. The genome was assembled by applying a *de novo* approach tailored with a scaffolding step on the OsHV-1 reference genome (AY509253), which allowed us to produce five large contigs ranging in length from 2684 to 164 511 bp and to merge them into a continuous sequence with three short ‘N’ stretches (64 ‘N’ bases in total), two of which were subsequently resolved by Sanger sequencing. The remaining ‘N’ stretch could not be resolved and its length was estimated solely on the basis of the scaffolding step. The final assembly was 203 983 bp long, with 1 ‘N’ stretch of 26 nucleotides (details are provided in the Supplementary Material).

The assembled OsHV-1-PT sequence showed a nucleotide composition of 38.6 % G+C, which is comparable to that of the reference OsHV-1 genome (38.7 %) and to the recently sequenced OsHV-1 µVar genome KY271630 (38.9 %), with 99.80 % and a 99.92 % nucleotide sequence similarity to the reference OsHV-1 and the OsHV-1 µVar sequences, respectively.

The OsHV-1-PT genome displayed the same structure as OsHV-1 µVar [13], with an organization that can be represented as TR_L_–U_L_–IR_L_–X–IR_S_–U_S_–TR_S_–X′ or X′–TR_L_–U_L_–IR_L_–X–IR_S_–U_S_–TR_S_, due to the impossibility of placing the X region exactly. OsHV-1-PT is characterized by the five large deletions and the large insertion discriminating the OsHV-1 µVar from the OsHV-1 reference [13]. The latter insertion had previously been detected in both the acute viral necrosis virus (ANVN) and *Scapharca broughtonii* ostreid herpesvirus-1 (OsHV-1-SB) genomes [19, 20]. The 86 bp insertion, found in the OsHV-1 µVar genome when compared to the OsHV-1 reference, was missing in OsHV-1-PT genome; instead, in the inverted repeat IR_S_/TR_S_ we found two additional deletions of 115 (starting at nucleotide 191 861 of IR_S_ and at nucleotide 199 578 of TR_S_) and 235 bp (starting at nucleotide 192 125 of IR_S_ and at nucleotide 200 107 of TR_S_). The assembled OsHV-1-PT shared 122 indels with the OsHV-1 µVar genomes, accounting for 1363 nucleotides, the majority of which (82.8 %) were short in length (<10 bp). The localization of most indels (82 %) in repeated sequence motifs (TR/IR) is not surprising as the performance of the *de novo* approach is well known to be difficult to apply on repeat-containing regions.

The open reading frame (ORF) prediction resulted in 125 different putative OsHV-1-PT proteins, including 111 unique ORFs and other 14 ORFs that were repeated twice in the genome because located in the IR regions (Table 2). We compared the ORFs of OsHV-1-PT (ORF^PT^) with those already described for OsHV-1 µVar (ORF^V^). The previously described indels led to the shortening of 4 ORFs (ORF^PT^ 3; 16; 114 and IN.4) down to 40 codons (Table 3), while 3 ORFs^PT^, namely 90, 119 and IN.1, gained 56, 43 and 137 extra amino acids, respectively. Sixty-one nucleotide substitutions, mostly non-synonymous (67.2 %), were found to be evenly distributed among 41 ORFs^PT^. Seven new ORFs were predicted and newly named from PT1–PT7. The remaining 70 predicted proteins were found to be completely conserved when compared to the ORFs of OsHV-1 µVar with the GenBank accession number KY271630 (blastn E-value=0 and similarity=100 %).

**Table 2.** ORF annotation and gene ontology classification for the µvar OsHV-1-PT For each putative ORF, information regarding the genomic coordinates (start, end), strand (positive or negative) and, if they are present, gene ontology, EC number and InterPro GO is reported. The column ‘Repeated’ indicates whether the ORF is present in repeated regions, and therefore has an alternative start, end and strand. Putative ORFs called ‘ORFnumber’ correspond to proteins in the reference (GenBank ID: AY509253); putative ORFs called ‘ORFIN.number’ are proteins that are partially or completely within an insertion; and putative ORFs called ‘ORF-PTnumber’ are new proteins that have been predicted and are not present in the reference

**Sequence**	**Start**	**End**	**Strand**	**Repeated**	**Description**	**GO IDs**	**GO names**	**EC IDs**	**EC names**	**InterPro GO IDs**	**InterPro GO names**
ORF1	77; 178159	523; 178605	Negative; positive	Yes	ORF1						
ORF2	641; 177538	1144; 178041	Negative; positive	Yes	ORF2						
ORF3	1845; 176073	2609; 176837	Positive; negative	Yes	ORF3						
ORF4	3336; 174297	4385; 175346	Negative; positive	Yes	ORF4						
ORF6	6361	8391	Negative	No	ORF6						
ORF7	8568	12113	Positive	No	DNA replication origin-binding helicase	F:GO:0016779; P:GO:0009058; C:GO:0005694; P:GO:0006259; C:GO:0005730	F:nucleotidyltransferase activity; P:biosynthetic process; C:chromosome; P:DNA metabolic process; C:nucleolus	EC: 2.7.7.6	DNA-directed RNA polymerase	F:GO:0003896; P:GO:0006269	F:DNA primase activity; P:DNA replication, synthesis of RNA primer
ORF8	12152	13111	Negative	No	ORF8						
ORF9	13217	14977	Negative	No	RING finger	F:GO:0043167	F:ion binding				
ORF10	15237	16265	Positive	No	DNA polymerase						
ORF11	16530	18431	Positive	No	Ribosomal L5						
ORF12	18537	19124	Positive	No	ORF12						
ORF13	19173	19490	Negative	No	Hypothetical protein						
ORF14	20042	20626	Positive	No	ORF14						
ORF15	20973	21617	Positive	No	Hypothetical protein						
ORF16	21698	21922	Positive	No	ORF16						
ORF17	22076	22423	Positive	No	ORF17						
ORF18	22475	22756	Positive	No	ORF18						
ORF19	22802	24010	Negative	No	ORF19						
ORF20	24090	25829	Negative	No	Ribonucleoside-diphosphate reductase subunit M2	P:GO:0044281; F:GO:0016491; F:GO:0043167; C:GO:0005829; P:GO:0009058; P:GO:0006259; P:GO:0044711	P:small-molecule metabolic process; F:oxidoreductase activity; F:ion binding; C:cytosol; P:biosynthetic process; P:DNA metabolic process; P:single-organism biosynthetic process	EC: 1.17.4; EC: 1.17.4.1	Acting on CH or CH(2) groups; ribonucleoside-diphosphate reductase	P:GO:0009263; P:GO:0055114	P:deoxyribonucleotide biosynthetic process; P:oxidation-reduction process
ORF21	25900	28854	Negative	No	ORF21						
ORF22	29018	33916	Positive	No	ORF22	C:GO:0005575	C:cellular_component				
ORF23	34047	37865	Negative	No	ORF23						
ORF24	37962	39095	Negative	No	ORF24						
ORF25	39178	39843	Negative	No	Y025_OSHVF AME: full=transmembrane ORF25 FLAGs: precursor	C:GO:0005575	C:cellular_component				
ORF26	39891	41078	Positive	No	ORF26						
ORF27	41188	41988	Negative	No	ORF27	P:GO:0044281; P:GO:0034641; F:GO:0003674	P:small molecule metabolic process; P:cellular nitrogen compound metabolic process; F:molecular_function				
ORF28	42073	44634	Negative	No	ORF28	C:GO:0016020; C:GO:0016021; P:GO:0046080; F:GO:0016787	C:membrane; C:integral component of membrane; P:dUTP metabolic process; F:hydrolase activity				
ORF29	44324	44929	Negative	No	ORF29						
ORF30	45032	45778	Negative	No	ORF30						
ORF31	45832	46389	Negative	No	ORF30						
ORF32	47184	48842	Positive	No	Y088_OSHVF AME: full=transmembrane ORF88 FLAGs: precursor	C:GO:0016020; C:GO:0016021; C:GO:0033644	C:membrane; C:integral component of membrane; C:host cell membrane				
ORF33	49157	49744	Negative	No	ORF33						
ORF34	49824	50198	Negative	No	ORF34						
ORF35	50284	50874	Negative	No	ORF35	C:GO:0016020; C:GO:0016021	C:membrane; C:integral component of membrane				
ORF38	50709	51266	Negative	No	RING finger	F:GO:0043167; C:GO:0005575	F:ion binding; C:cellular_component				
ORF39	51388	51972	Negative	No	ORF38						
ORF40	51975	53702	Negative	No	ORF40						
ORF41	53853	56774	Positive	No	Hypothetical protein	C:GO:0005575	C:cellular_component				
ORF42	56811	57905	Negative	No	E3 ubiquitin ligase XIAP-like	F:GO:0043167	F:ion binding				
ORF43	58023	58634	Positive	No	ORF43						
ORF44	61657	62583	Negative	No	ORF44						
ORF45	62727	63452	Positive	No	ORF45						
ORF46	63600	64247	Positive	No	ORF46						
ORF47	64263	68501	Positive	No	ORF47						
ORF49	68707	72123	Positive	No	DNA replication origin-binding helicase	F:GO:0003896; F:GO:0003688; F:GO:0005524; P:GO:0006260; F:GO:0004386; F:GO:0016740; P:GO:0006269	F:DNA primase activity; F:DNA replication origin binding; F:ATP binding; P:DNA replication; F:helicase activity; F:transferase activity; P:DNA replication, synthesis of RNA primer				
ORF50	73476	74777	Negative	No	na						
ORF51	75289	77796	Negative	No	Ribonucleoside-diphosphate reductase large subunit	P:GO:0044281; F:GO:0016491; F:GO:0043167; C:GO:0005829; P:GO:0009058; P:GO:0006259	P:small molecule metabolic process; F:oxidoreductase activity; F:ion binding; C:cytosol; P:biosynthetic process; P:DNA metabolic process	EC:1.17.4; EC:1.17.4.1	Acting on CH or CH(2) groups; ribonucleoside-diphosphate reductase	F:GO:0005524; F:GO:0004748; P:GO:0006260; P:GO:0055114	F:ATP binding; F:ribonucleoside-diphosphate reductase activity, thioredoxin disulfide as acceptor; P:DNA replication; P:oxidation-reduction process
ORF52	77913	78455	Positive	No	ORF52						
ORF53	78539	80086	Positive	No	ORF53						
ORF54	80145	82568	Positive	No	Y068_OSHVF AME: full=transmembrane ORF68 FLAGs: precursor	C:GO:0005575	C:cellular_component				
ORF55	82645	83064	Negative	No	ORF55						
ORF56	83338	84186	Positive	No	ORF56						
ORF57	83936	84886	Positive	No	Chloride channel CLIC 1	C:GO:0005737; C:GO:0043226	C:cytoplasm; C:organelle				
ORF58	84932	86497	Positive	No	ORF58						
ORF59	86590	89832	Positive	No	Hypothetical protein	C:GO:0005575	C:cellular_component				
ORF60	89885	91120	Negative	No	ORF60						
ORF61	91298	93034	Positive	No	ORF61						
ORF64	93453	94649	Negative	No	RNA ligase	F:GO:0016874	F:ligase activity				
ORF66	96797	100189	Negative	No	ORF66	F:GO:0016779; P:GO:0009058; C:GO:0005694; P:GO:0006259; C:GO:0005730	F:nucleotidyltransferase activity; P:biosynthetic process; C:chromosome; P:DNA metabolic process; C:nucleolus	EC:2.7.7.6	DNA-directed RNA polymerase	F:GO:0003896; P:GO:0006269	F:DNA primase activity; P:DNA replication, synthesis of RNA primer
ORF67	100603	102393	Positive	No	DEAD-box ATP-dependent RNA helicase	F:GO:0003677; F:GO:0005524; F:GO:0004386; F:GO:0016787	F:DNA binding; F:ATP binding; F:helicase activity; F:hydrolase activity				
ORF68	102474	104555	Negative	No	Y068_OSHVF AME: full=transmembrane ORF68 FLAGs: precursor	C:GO:0005575	C:cellular_component				
ORF69	104623	106014	Negative	No	ORF69						
ORF70	106498	107100	Negative	No	ORF70						
ORF71	107387	108748	Positive	No	ORF71						
ORF72	108646	109212	Positive	No	ORF72	C:GO:0005575	C:cellular_component				
ORF74	111208	111564	Negative	No	ORF74						
ORF75	111620	112330	Negative	No	ORF75	P:GO:0044281; P:GO:0034641; F:GO:0003674	P:small molecule metabolic process; P:cellular nitrogen compound metabolic process; F:molecular_function				
ORF76	113161	115197	Positive	No	ORF75						
ORF77	115315	119109	Positive	No	Hypothetical protein	C:GO:0005575	C:cellular_component				
ORF78	119163	122618	Positive	No	ORF78						
ORF79	122633	123073	Positive	No	ORF79						
ORF80	123137	123487	Positive	No	Hypothetical protein	C:GO:0005575	C:cellular_component				
ORF81	123611	124252	Positive	No	ORF81						
ORF82	124200	125090	Positive	No	ORF82						
ORF83	125185	126291	Negative	No	ORF83						
ORF84	126297	126653	Negative	No	ORF83	C:GO:0005575	C:cellular_component				
ORF85	126658	128661	Positive	No	ORF85						
ORF86	128665	129072	Positive	No	ORF86						
ORF87	129087	129599	Positive	No	E3 ubiquitin ligase XIAP	F:GO:0008270; F:GO:0046872; P:GO:0042981	F:zinc ion binding; F:metal ion binding; P:regulation of apoptotic process				
ORF88	129693	131939	Positive	No	Y088_OSHVF AME: full=transmembrane ORF88 FLAGs: precursor	C:GO:0005575	C:cellular_component				
ORF89	131991	132725	Positive	No	ORF89						
ORF90	132786	133838	Negative	No	ORF89						
ORF91	133786	134868	Negative	No	ORF91	C:GO:0016020; C:GO:0016021	C:membrane; C:integral component of membrane				
ORF92	134917	135606	Negative	No	ORF92						
ORF93	135542	136756	Negative	No	ORF93						
ORF94	136761	137804	Positive	No	ORF94	F:GO:0008168; P:GO:0006259; P:GO:0006950	F:methyltransferase activity; P:DNA metabolic process; P:response to stress	EC:2.1.1.63	Methylated-DNA–[protein]-cysteine S-methyltransferase	P:GO:0006281; F:GO:0003908	P:DNA repair; F:methylated-DNA-[protein]-cysteine S-methyltransferase activity
ORF95	137889	138821	Positive	No	Exonuclease V	F:GO:0003677; F:GO:0004518; P:GO:0034655; P:GO:0006259	F:DNA binding; F:nuclease activity; P:nucleobase-containing compound catabolic process; P:DNA metabolic process	EC:3.1.11; EC:3.1.15	Acting on ester bonds; Acting on ester bonds	F:GO:0003677; F:GO:0045145; F:GO:0004518	F:DNA binding; F:single-stranded DNA 5′−3′ exodeoxyribonuclease activity; F:nuclease activity
ORF96	138886	139608	Negative	No	E3 ubiquitin ligase LRSAM1	F:GO:0043167	F:ion binding				
ORF97	139689	140234	Negative	No	E3 ubiquitin ligase LRSAM1	F:GO:0043167	F:ion binding				
ORF98	140737	142491	Positive	No	ORF98						
ORF99	142863	143615	Negative	No	Inhibitor of apoptosis	F:GO:0008270; F:GO:0046872; P:GO:0016032; P:GO:0039526	F:zinc ion binding; F:metal ion binding; P:viral process; P:modulation by virus of host apoptotic process				
ORF100	144107	149743	Positive	No	DNA polymerase delta catalytic	F:GO:0003677; F:GO:0016779; F:GO:0004518; P:GO:0009058; P:GO:0006259; C:GO:0005622	F:DNA binding; F:nucleotidyltransferase activity; F:nuclease activity; P:biosynthetic process; P:DNA metabolic process; C:intracellular	EC:2.7.7.7	DNA-directed DNA polymerase	F:GO:0003676; F:GO:0000166; F:GO:0008408; F:GO:0003677; F:GO:0003887	F:nucleic acid binding; F:nucleotide binding; F:3′−5′ exonuclease activity; F:DNA binding; F:DNA-directed DNA polymerase activity
ORF101	149830	150459	Positive	No	ORF101						
ORF102	150499	152787	Negative	No	ORF102						
ORF103	152800	154071	Positive	No	Y103_OSHVF AME: full=transmembrane ORF103	C:GO:0005575	C:cellular_component				
ORF104	154220	157822	Positive	No	ORF104						
ORF106	159718	161115	Negative	No	Inhibitor of apoptosis 1	F:GO:0043167	F:ion binding				
ORF107	161239	163308	Positive	No	ORF107						
ORF108	163525	164337	Positive	No	ORF108						
ORF109	164390	167014	Positive	No	DNA packaging terminase subunit 1 [Felid alphaherpesvirus 1]	P:GO:0051276	P:chromosome organization			P:GO:0006323	P:DNA packaging
ORF110	167109	167894	Positive	No	ORF110						
ORF111	167983	168852	Negative	No	Y111_OSHVF AME: full=transmembrane ORF111	C:GO:0005575	C:cellular_component				
ORF112	168976	170364	Positive	No	ORF112						
ORF113	170371	171327	Positive	No	ORF113						
ORF114	171473	172321	Positive	No	ORF6						
ORF115	178794;203136	179555;203897	Positive; negative	Yes	ORF115	F:GO:0003677; F:GO:0043167; P:GO:0009058; P:GO:0006259; C:GO:0005575	F:DNA binding; F:ion binding; P:biosynthetic process; P:DNA metabolic process; C:cellular_component				
ORF116	181293;200628	182063;201398	Positive; negative	Yes	ORF116						
ORF117	182787;198789	183902;199904	Negative; positive	Yes	RING finger	F:GO:0043167	F:ion binding				
ORF118	184136;197887	184804;198555	Negative; positive	Yes	RING finger	F:GO:0043167	F:ion binding				
ORF119	185173;196940	185751;197518	Positive; negative	Yes	ORF119						
ORF120	186193;196175	186516;196498	Positive; negative	Yes	ORF120						
ORF121	186637;195407	187284;196054	Negative; positive	Yes	RING finger	F:GO:0043167	F:ion binding				
ORF122	187985;193552	189139;194706	Negative; positive	Yes	ORF122						
ORF123	189857	190768	Negative	No	ORF123						
ORF124	191371	192795	Negative	No	ORF124						
ORFIN.1	58692	59627	Positive	No	ORF125						
ORFIN.3	60105	60578	Positive	No	ORF126						
ORFIN.4	60601	61521	Positive	No	ORF127	C:GO:0005575	C:cellular_component				
ORF-PT1	4503;173442	5240;174179	Negative; positive	Yes	Hypothetical protein	C:GO:0016020; C:GO:0016021; C:GO:0033644	C:membrane; C:integral component of membrane; C:host cell membrane				
ORF-PT2	5765;172540	6142;172917	Negative; positive	Yes	na						
ORF-PT3	106114	106497	Positive	No	na						
ORF-PT4	109747	110373	Positive	No	na						
ORF-PT5	110433	111110	Positive	No	na						
ORF-PT6	157959	158690	Negative	No	na						
ORF-PT7	158653	159138	Negative	No	Hypothetical protein AbHV_ORF44						

**Table 3.** Differences between ORFs found in OsHV-1 µvar-PT and OsHV-1 µVar (KY271630, [13])

	OsHV-1 µvar-PT		OsHV-1 (KY271630)			
ORF ID	Length (bp)	Length (codons)	Nucleotide similarity	No. of SNP, nsSNP	Length difference (AA)	Function/family/domain
ORF1	447	149	99.55	2, 1	0	Unknown
ORF2	504	168	99.8	1, 1	0	Unknown
ORF3	765	255	99.35	2, 1	−1	Unknown
ORF4	1050	350	99.81	2, 2	0	Unknown
ORF6	2031	677	99.95	1, 0	0	Unknown
ORF7	3546	1182	99.94	2, 2	0	Putative helicase
ORF11	1902	634	99.89	2, 1	0	Unknown
ORF16	225	75	97.4	1, 1	−2	Membrane protein
ORF17	348	116	99.71	1, 1	0	Secreted
ORF20	1740	580	99.94	1, 1	0	Ribonucleotide reductase small subunit
ORF21	2955	985	99.97	1, 1	0	Unknown
ORF22	4899	1633	99.98	1, 1	0	Transmembrane protein
ORF23	3819	1273	99.97	1, 1	0	Unknown
ORF28	2562	854	99.96	1, 1	0	Unknown
ORF32	1659	553	99.82	1, 1	0	Transmembrane glycoprotein
ORF34	375	125	99.73	1, 1	0	Unknown
ORF41	2922	974	99.9	1, 1	0	Transmembrane protein
ORF43	612	204	99.84	1, 0	0	Unknown
ORF47	4239	1413	99.93	3, 1	0	Unknown
ORF49	3417	1139	99.91	1, 1	0	Unknown
ORF50	1302	434	100	1, 0	0	Unknown
ORF59	3243	1081	99.97	1, 1	0	Transmembrane glycoprotein
ORF68	2082	694	99.95	1, 0	0	Transmembrane protein
ORF71	1362	454	99.93	1, 0	0	Unknown
ORF76	2037	679	99.9	2, 2	0	Unknown
ORF77	3795	1265	99.97	1, 1	0	Transmembrane glycoprotein
ORF78	3456	1152	99.94	2, 1	0	Unknown
ORF80	351	117	99.72	1, 0	0	Transmembrane glycoprotein
ORF88	2247	749	99.82	4, 4	0	Transmembrane glycoprotein
ORF89	735	245	99.86	1, 0	0	Unknown
ORF90	1053	351	100	0	56	Unknown
ORF94	1044	348	99.9	1, 1	0	Unknown
ORF98	1755	585	99.94	1, 0	0	Unknown
ORF100	5637	1879	99.96	2, 2	0	Catalytic subunit DNA polymerase
ORF101	630	210	99.84	1, 0	0	Unknown
ORF106	1398	466	99.93	1, 0	0	Zinc-finger, ring type, BIR domain
ORF107	2070	690	99.9	2, 2	0	Unknown
ORF112	1389	463	99.93	1, 0	0	Unknown
ORF114	849	283	100	0	−40	Unknown
ORF115	762	254	99,.48	2, 1	0	Replication origin-binding protein
ORF116	771	257	99.48	4, 4	0	Unknown
ORF119	579	193	99.33	0	43	Unknown
ORF120	324	108	99.69	1, 1	0	Secreted
ORF121	648	216	99.85	1, 0	0	Zinc-finger, ring type
ORF123	912	304	99.78	2, 1	0	Unknown
ORF124	1425	475	99.79	3, 2	0	Unknown
ORF IN.1	936	312	99.68	0	137	Secreted
ORFIN.4	921	307	98.35	2, 2	−20	Transmembrane glycoprotein, 1 helix
ORF-PT1	738	246	\	\	\	Membrane protein
ORF-PT2	375	126	\	\	\	Unknown
ORF-PT3	378	128	\	\	\	Unknown
ORF-PT4	384	209	\	\	\	Unknown
ORF-PT5	378	226	\	\	\	Unknown
ORF-PT6	627	244	\	\	\	Unknown
ORF-PT7	375	162	\	\	\	Unknown

All putative ORFs were functionally annotated using the NCBI NR protein database, Gene Ontology (GO) [26, 27] and the Kyoto Encyclopedia of Genes and Genomes (Table 2). It was possible to assign definitions [28] to 119 of the ORFs (95.2 %) by homology search. We also assigned GO terms to 45 ORFs (36 %), Enzyme Commission numbers and InterPro GO terms to 7 and 8 ORFs, respectively. As a result, it was possible to assign a definition to two of the seven new predicted ORFs (PT1–PT7) and to assign GO terms to PT1, revealing its putative function as an integral membrane protein.

To better investigate the genotype of the Italian OsHV-1-PT, a phylogenetic analysis based on the C region, currently regarded as the most variable region, was performed according to previously published studies [4, 12]. The OsHV-1-PT sequence of the C region, including ORFs 4/5, was compared with all OsHV-1 sequences retrieved from GenBank and representing different geographical areas. As expected, all the OsHV-1 microvariant sequences and OsHV-1-PT clustered together, although with a bootstrap value lower than 70 (data not shown). The progressive whole-genome sequencing of new *Malacoherpesviridae* viruses should produce a more refined phylogenetic classification and provide support the functional characterization of the OsHV-1 variants currently affecting bivalve hosts.

In conclusion, we demonstrated that the next generation sequencing and subsequent *de novo* assembly approach represent a valid strategy for reconstructing the genome of a dsDNA virus, such as OsHV-1, with high-confidence, even in case of non-enriched, unpurified samples at relatively low sequencing depth. The availability of *Malacoherpesviridae* genomes can lead to a real understanding of functional virus features, i.e. the identification of virulence factors in OsHV-1 variants, as well as phylogenetic relationships and the evolutionary origin of mollusk viruses. Owing to the reported sequence features, we propose the Porto Tolle OsHV-1 virus as a new microvariant. Needless to say, additional studies that relate the pathogenic occurrence of OsHV-1 to developmental stages and environmental conditions are needed to fully characterize the pathogenicity of the Italian OsHV-1-PT virus.

## Supplementary Data

731 Supplementary File 1
