# Supplementary material for: Identification of a newly described OsHV-1 µvar from the North Adriatic Sea (Italy)
Source: J Gen Virol. 2018 Mar 26;99(5):693–703. doi: 10.1099/jgv.0.001042 (PMC5994699; doi:10.1099/jgv.0.001042)
Supplement: Supplementary File 1 [file jgv-99-693-s001.pdf]

Materials and methods

DNA extraction. Total DNA was purified from oyster tissue fragments (gills and mantle) using the QIAamp DNA Mini Kit® according to the manufacturer's instructions (Qiagen, Venlo, the Netherlands). The DNA samples were eluted in 50 µl of elution buffer supplied by Qiagen, quantified using the NanoDrop™ spectrophotometer (ThermoFisher Scientific™, Waltham, MA, USA) and stored at -20°C.

qPCR protocol (OsHV-1). After dilution to 5 ng μl^{-1}, 5 µl of each individual DNA sample was added to the quantitative real-time PCR reaction mix composed of 12.5 µl SsoFast™ EvaGreen® Supermix (Bio-Rad), 1.25 µl of both HVDP-F (5' ATTGATGATGTGGATAATCTGTG 3') and HVDP-R (5' GGTAAATACCATTGGTCTTGTTCC 3') primers targeting the catalytic subunit of the viral DNA polymerase (ORF100, nucleotides 147655-153291 of the reference OsHV-1 genome AY509253[1]), each diluted at the concentration of 0.5 µM, and 5 µl of water. All amplification reactions were performed in a Rotor-Gene® Q thermocycler (Qiagen) as follows: 1 cycle of polymerase activation at 95◦C for 5 min; 40 cycles of amplification at 95°C for 30 s, 60°C for 1min, and 72°C for 45 s and a final step for melting temperature curve analysis from 65 to 95°C (10 sec/step, ramp rate 0.5°C/sec) [2].

Absolute quantification of copies of OsHV-1 DNA (copies μl^{-1}) was carried out by comparing C_t values obtained with a standard curve, using the Rotor-Gene® Q thermocycler software. The standard curve was prepared using plasmidic DNA, corresponding to the OsHV-1 target region, serially diluted 1:10 in triplicate (10 to 10^6 DNA copies μl^{-1}) and also used as positive control.

Inoculum preparation. Starting from the naturally infected oysters, all inocula were freshly prepared from oysters showing viral DNA loads above 10^6 copies μl^{-1}, as assessed by OsHV-1-specific qPCR. Briefly, each oyster was opened by removing the superior valve, gills and mantle were dissected and homogenized with mortar, pestle and quartz powder. Each homogenate was then centrifuged at 4000 rpm for 30 minutes at 4°C and all the supernatants were stored at 4°C until the infection.

Experimental infection. Native *Magallana gigas* oysters of about 4 cm shell length, tested free from OsHV-1 using the qPCR protocol described above, were acclimated for at least 10 days in a 280 L tank (Instant Ocean seawater reconstituted at 33 psu; 21°C; 6 ppm of dissolved O_2; artificial light for 10 h d^{-1}; commercial feed for invertebrates supplied on alternate days). After injection of a OsHV-1-positive inoculum or sterile seawater, the treated oysters were moved to a 50 L tank and monitored for a period of 6-7 days (same standard conditions). Precisely, after preliminary anesthesia in magnesium chloride-enriched seawater (4 h in 25 g $MgCl_2$ l^{-1} SW) [3] up to 13 oysters per infection trial (total 145) were individually injected into the relaxed adductor muscle with 100-150 µl of inoculum, depending on the amount and viral titer of the supernatant recovered each time from virus-positive oysters. Daily mortality was recorded and dead/moribund oysters were systematically removed. Both dead and survived oysters collected at the end of each infection trial were stored at -80°C for further processing. A piece of both gill and mantle (25 mg w.w. tissue) was sampled from each oyster to assess the individual viral concentration by the qPCR described above.

OsHV-1-PT and *M.gigas* DNA ratio. In order to calculate the genomic copy ratio OsHV-1-PT / *M. gigas*, we quantified OsHV-1 DNA and *M. gigas* DNA employing two distinct qPCR protocols on the same sample. The qPCR protocol used for OsHV-1 is described above. Oyster DNA quantification was carried out via a qPCR targeting the *M. gigas* gene EF1α (elongation factor-1α, primer forward GCATTTTGGTGCCTCTTCCA, primer reverse ACCACCCTGGTGAGATCAAG). Briefly, real-time PCR analysis was conducted in 25 µl containing 0.5 µM of each primer, 12.5 µl SsoFast™ EvaGreen® Supermix (Bio-Rad) and 5 µl of sample DNA. All amplification reactions were performed in a Rotor-Gene® Q thermocycler (Qiagen) as follows: 1 cycle of polymerase activation at 95◦C for 5 min; 40 cycles of amplification at 95°C for 30 s, 60°C for 1min, and 80°C for 15 s (fluorescence acquisition step) and a final step for melting temperature curve analysis from 65 to 95°C (10 sec/step, ramp rate 0.5°C/sec). Each qPCR protocol was applied on 5 log10 serial dilutions of sample DNA, prepared in triplicate from of an oyster highly infected with OsHV-1-PT selected for subsequent DNA

sequencing. The respective regression curves were then analyzed to demonstrate their parallelism, a condition necessary to calculate the ΔCt value (Ct EF1α – Ct OsHV-1). Such value was employed to calculate the relative fold concentration by the formula $2^{-\Delta Ct}$

Statistical analysis. The cumulative Kaplan-Meier curve was plotted with the survfit function implemented in the survival package [4] under the R software environment [5].

Library preparation. Sequencing library, starting from the genomic DNA of the selected sample, was prepared using Nextera XT DNA sample preparation kit (Illumina) and according to the manufacturer's instructions, with only minor modifications. Library was quantified with the Qubit Fluoremeter using the Qubit DNA HS Assay Kit (Thermo Fisher) whereas quality and fragments size were inspected using Agilent High Sensitivity DNA kit (Agilent). The library was processed on an Illumina Miseq desktop sequencer using Miseq v3 Reagent Kit (300PE).

Data preprocessing. Illumina reads quality was assessed using FastQC v0.11.2 [6]. Raw data were filtered by removing: a) reads with more than 10% of undetermined ("N") bases; b) reads with more than 100 bases with Q score below 7; c) duplicated paired-end reads. Remaining reads were clipped from Illumina adaptors Truseq with scythe v0.991 (https://github.com/vsbuffalo/scythe) and trimmed with sickle v1.33 (https://github.com/najoshi/sickle). Reads shorter than 80 bases or unpaired after previous filters were discarded.

Metagenomic analysis. Taxonomic assignment of high-quality host-free reads was carried out using BLASTN 2.6.0+ [7] alignment against the integrated NT database (version 8 February 2017) and diamond v0.8.36 [8] alignment against the integrated NR database (version 8 February 2017). Alignment hits with e-values greater than 1×10^{-3} were filtered. Taxonomical level of each read were determined by the lowest common ancestor (LCA)-based algorithm that was implemented in MEGAN v6.7.0 [9].

***De novo* assembly.** For the reconstruction of OsHV-1 consensus sequence, reads taxonomically classified as belonging to *Herpesvirales* order were selected and *de novo* assembled using IDBA-UD v1.1.1 [10] with the multi-kmer approach using a minimum value of 24, a maximum value of 124 and an inner increment of 5. Order, orientation and repeats of contigs were determined by aligning them with MUMmer v3.1 [11] against OsHV-1 reference genome (GenBank: AY509253). Based on this alignment, contigs were linked with an appropriate stretch of "N" bases, if required, thus producing the consensus sequence of OsHV-1 genome.
In order to assure that the consensus sequence was truly representing the OsHV-1 genome present in the sample, we aligned all reads classified as belonging to *Herpesvirales* against the consensus sequence with BWA v0.7.12-r1039 [12]. We performed a visual inspection of the alignment with tablet v1.14.10.21 [13] and manually revised the consensus sequence based on this alignment. We also checked whether all positions in the consensus sequence were filled with the consensus nucleotide at that position by calling variants with LoFreq v2.1.2 [14]. According to LoFreq usage recommendations, the alignment was first processed with Picard-tools v2.1.0 (http://picard.sourceforge.net) and GATK v3.5 [15-17] in order to correct potential errors, realign reads around indels and recalibrate base quality. LoFreq was then run on fixed alignment with option "--call-indels" to produce a vcf files containing both SNPs and indels. From the final set of variants indels and SNPs with a frequency lower than 50% were discarded. If needed, we used remaining variants to accordingly change OsHV-1 consensus sequence.

Annotation. Putative open reading frames (ORFs) were identified using the NCBI ORF finder(https://www.ncbi.nlm.nih.gov/orffinder/), setting a minimal length of 100 codons, according to the criteria described by Davison *et al.* (2005) and commonly used for the annotation of all *Malacoherpesviridae* genomes. Briefly, overlapping ORFs and ORFs shorter than the minimal length were considered if they displayed peculiar features supporting their real existence, such as conserved domain(s), transmembrane or signal peptide region. The ORFs were named in agreement with the previously published OsHV-1 genomes. The amino acid sequences were analyzed using NCBI BLASTP (https://www.ncbi.nlm.nih.gov/), by comparing OsHV-1-PT, the µVar and the reference genotypes [18],

[19]. Conserved Pfam protein domains were identified using HMMer and applying a 0.01 cut-off whereas signal peptide and transmembrane regions were identified with SignalP v. 4.0 and TMHMM v. 2.0, respectively. Putative ORFs were functionally annotated with Blast2GO [20].

Gap closing PCR and Sanger sequencing. Different sets of primers were designed according to the "N" base positions. The PCRs were carried out using a Platinum Taq DNA polymerase kit (Invitrogen) according to the manufacturer's instructions and running the PCR amplification products in 1% agarose gel. The PCR products were purified with ExoSAP-IT® (USB Corporation, Cleveland, OH) and sequenced in both directions using the Big Dye Terminator v3.1 cycle sequencing kit (Applied Biosystems, Foster City, CA). The sequencing reactions were cleaned-up using CENTRI-SEP 96 Well Plates (Princeton Separations, Inc.) according to the manufacturer's instructions and analyzed on a 16-capillary ABI PRISM 3130xl Genetic Analyzer (Applied Biosystems, Foster City, CA, USA). Sequencing data were assembled and edited using dedicated software as Sequencing Analysis 5.2 and SeqScape v2.5 (Applied Biosystems).

Reference

[1] **Webb SC, Fidler A, Renault T**. Primers for PCR-based detection of ostreid herpes virus-1 (OsHV-1): Application in a survey of New Zealand molluscs. *Aquaculture* 2007; 272:126–139.

[2] **Segarra A, Baillon L, Faury N, Tourbiez D, Renault T.** Detection and distribution of ostreid herpesvirus 1 in experimentally infected Pacific oyster spat. *J Invertebr Pathol* 2016; 133:59-65.

[3] **Suquet M, de Kermoysan G, Araya RG, Queau I, Lebrun L, Le Souchu P, Mingant C.** Aquatic Living Resources Anesthesia in Pacific oyster, Crassostrea gigas. *Aquat Living Resour* 2006; 22:29–34.

[4] **Therneau TM.** A Package for Survival Analysis in S. 2015.

[5] **R Core Team.** R: A Language and Environment for Statistical Computing. 2016.

[6] **Andrews S.** FastQC, A quality control tool for high throughput sequence data. http://www.bioinformatics.babraham.ac.uk/projects/fastqc. 2014.

[7] **Altschul SF, Gish W, Miller W, Myers EW, Lipman DJ.** Basic local alignment search tool. *J Mol Biol* 1990; 215:403–410.

[8] **Buchfink B, Xie C, Huson DH.** Fast and sensitive protein alignment using DIAMOND. *Nat Methods* 2014; 12:59–60.

[9] **Huson DH, Beier S, Flade I, Górska A, El-Hadidi M, Mitra S, Ruscheweyh HJ, Tappu R.** MEGAN Community Edition - Interactive Exploration and Analysis of Large-Scale Microbiome Sequencing Data. *PLoS Comput Biol* 2016; 12:1–12.

[10] Peng Y, Leung HCM, Yiu SM, Chin FYL. IDBA-UD: a de novo assembler for single-cell and metagenomic sequencing data with highly uneven depth. *Bioinformatics* 2012; 28:1420–1428.

[11] **Kurtz S, Phillippy A, Delcher AL, Smoot M, Shumway M, Antonescu C, Salzberg SL.** Versatile and open software for comparing large genomes. *Genome Biol* 2004; 5:R12.

[12] **Li H, Durbin R.** Fast and accurate long-read alignment with Burrows–Wheeler transform. *Bioinformatics* 2010; 26:589–595.

[13] **Milne I, Bayer M, Cardle L, Shaw P, Stephen G, Wright F, Marshall D.** Tablet--next generation sequence assembly visualization. *Bioinformatics* 2010; 26:401–402.

[14] **Wilm A, Aw PP, Bertrand D, Yeo GH, Ong SH, Wong CH, Khor CC, Petric R, Hibberd ML, Nagarajan N.** LoFreq: a sequence-quality aware, ultra-sensitive variant caller for uncovering cell-population heterogeneity from high-throughput sequencing datasets. *Nucleic Acids Res* 2012; 40:11189–11201.

[15] **McKenna A, Hanna M, Banks E, Sivachenko A, Cibulskis K, Kernytsky A, Garimella K, Altshuler D, Gabriel S, Daly M, DePristo MA.** The Genome Analysis Toolkit: A MapReduce framework for analyzing next-generation DNA sequencing data. *Genome Res* 2010; 20:1297–1303.

[16] **DePristo MA, Banks E, Poplin R, Garimella KV, Maguire JR, Hartl C, Philippakis AA, del Angel G, Rivas MA, Hanna M, McKenna A, Fennell TJ, Kernytsky AM, Sivachenko AY, Cibulskis K, Gabriel SB, Altshuler D, Daly MJ.** A framework for variation discovery and genotyping using next-generation DNA sequencing data. *Nat Genet* 2011; 43:491–498.

[17] **Van der Auwera GA, Carneiro MO, Hartl C, Poplin R, Del Angel G, Levy-Moonshine A, Jordan T, Shakir K, Roazen D, Thibault J, Banks E, Garimella KV, Altshuler D, Gabriel S, DePristo MA.** From FastQ Data to High-Confidence Variant Calls: The Genome Analysis Toolkit Best Practices Pipeline. *Curr Protoc Bioinformatics* 2013; 43:11.10.1-11.10.33.

[18] **Burioli EAV, Prearo M, Houssin M.** Complete genome sequence of Ostreid herpesvirus type 1 µVar isolated during mortality events in the Pacific oyster Crassostrea gigas in France and Ireland. *Virology* 2017; 509:239–251.

[19] **Davison AJ, Trus BL, Cheng N, Steven AC, Watson MS, Cunningham C, Le Deuff RM, Renault T.** A novel class of herpesvirus with bivalve hosts. *J Gen Virol* 2005; 86:41–53.

[20] **Conesa A, Gotz S, Garcia-Gomez JM, Terol J, Talon M, Robles M.** Blast2GO: a universal tool for annotation, visualization and analysis in functional genomics research. *Bioinformatics* 2005; 21:3674–3676.

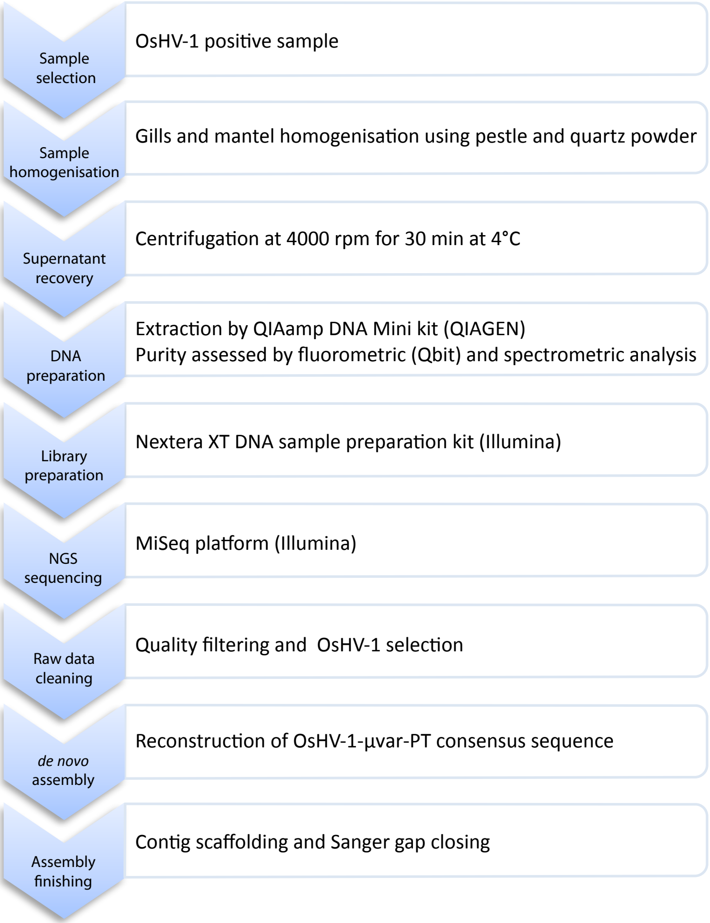

Supplementary Fig. 1. Description of OsHV-1-PT whole genome sequencing workflow

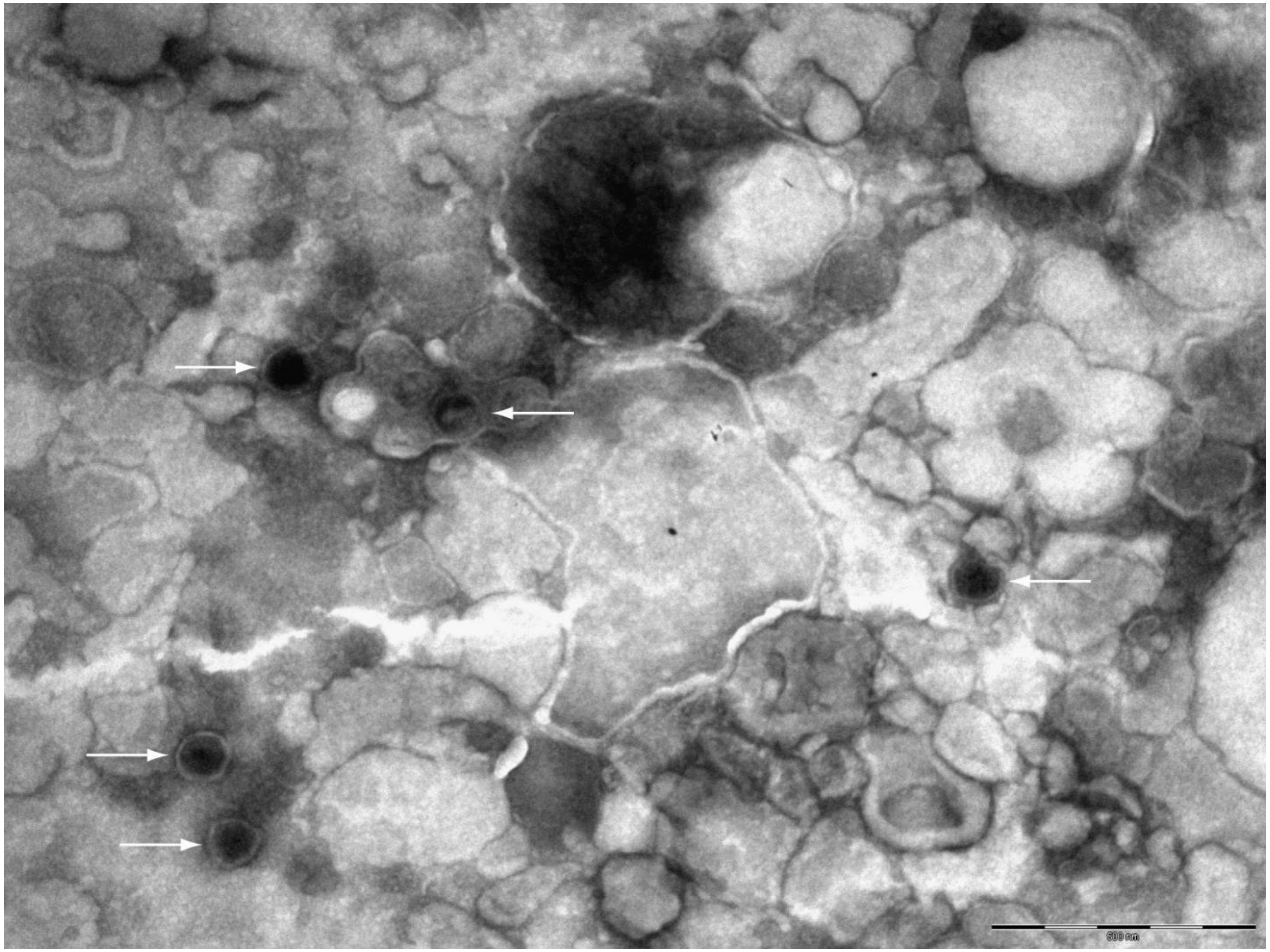

Supplementary Fig. 2 TEM image of herpesvirus-like particles in tissues from an OsHV-1-infected oyster. Negative staining, 36Kx magnification (bar 500 nm)
